# Emotion, emotion regulation and sleep: An intimate relationship

**DOI:** 10.3934/Neuroscience.2018.1.1

**Published:** 2017-12-01

**Authors:** Marie Vandekerckhove, Yu-lin Wang

**Affiliations:** 1 Faculty of Psychology and Educational Sciences, Vrije Universiteit Brussel, 1050 Etterbeek, Belgium; 2 Department of Data Analysis, Faculty of Psychological and Pedagogical Sciences, University of Gent, B-9000 Gent, Belgium

**Keywords:** sleep, stress, emotion, emotion regulation, modulation, rapid eye movement

## Abstract

In recent years, research has witnessed an increasing interest in the bidirectional relationship between emotion and sleep. Sleep seems important for restoring daily functioning, whereas deprivation of sleep makes us more emotionally aroused and sensitive to stressful stimuli and events. Sleep appears to be essential to our ability to cope with emotional stress in everyday life. However, when daily stress is insufficiently regulated, it may result in mental health problems and sleep disturbances too. Not only does emotion impact sleep, but there is also evidence that sleep plays a key role in regulating emotion. Emotional events during waking hours affect sleep, and the quality and amount of sleep influences the way we react to these events impacting our general well-being. Although we know that daytime emotional stress affects sleep by influencing sleep physiology, dream patterns, dream content and the emotion within a dream, its exact role is still unclear. Other effects that have been found are the exaggeration of the startle response, decrease in dream recall and elevation of awakening thresholds from rapid eye movement (REM), REM-sleep, increased or decreased latency to REM-sleep, increase in percentage of REM-density, REM-sleep duration, as well as the occurrence of arousals in sleep as a marker of sleep disruption. Equally, the way an individual copes with emotional stress, or the way in which an individual regulates emotion may modulate the effects of emotional stress on sleep. The research presented here supports the idea that adaptive emotion regulation benefits our follow-up sleep. We thus conclude the current review with a call for future research in order to clarify further the precise relationship between sleep, emotion and emotion regulation, as well as to explain further how sleep dissolves our emotional stress.

## 1. Introduction

The function of sleep within the realm of learning [1], memory [2], physical recovery [3], metabolism [4] and immunity [5] is well documented across species [6]. Abundant research attention has focused on the study of plausible causes of sleep disturbance (*e.g.*, insomnia [7]), such as emotional stress. The relationship between coping and sleep has also been a topic of recent research. In particular, recent research has revealed the function of sleep in regulating emotion to be quintessential [7]. A good night of sleep is crucial for mental and physical health and well-being. *Vice versa*, the ability of an individual to regulate emotion plays a vital role in decreasing the detrimental effects of emotional stress on sleep physiology [8],[9]. For instance, it has been shown how emotion dysregulation and affect are related to poor sleep quality [8]. In the current review, we will first briefly review the role of intact sleep in modulating emotion, and then follow this by discussing the effects of stress (*e.g.*, painful or emotional experiences) on sleep as well as how the regulation of emotion can impact sleep activity. In the discussion on how different styles of emotion regulation affect these emotionally stressful experiences [10], we will especially focus in particular on a recently investigated experiential approach of emotion regulation. For instance, we will discuss emotional approach coping (EAC) [11] in such a manner as to eliminate misunderstandings concerning emotion oriented coping, since these misunderstanding have clouded the research results for many years. Importantly, recent work has suggested the adaptive potential role of EAC in coping with both minor everyday stresses and with major stressful life challenges such as infertility, cancer, chronic pain and sleep. Therefore, we try to clarify whether and how this mode of emotion regulation plays a role in easing the detrimental effects of negative emotional experiences on our sleep physiology. Finally, we point to some important directions for future research.

## 2. Emotional processing role of sleep

Although different opinions and assumptions exist as to the exact functions of sleep, it is widely accepted that sleep plays an adaptive role in the processing of daily stressors and emotions. Sleep deprivation is followed by the rebound of rapid eye movement (REM) sleep and slow wave sleep (SWS) in the following nights [12],[13]. This effect suggests that a certain amount of REM-sleep and SWS is crucial in sleep. Clinical evidence suggests that sleep has a role in regulating our emotional brain-state [14] since sleep impairment corresponds to affective dysfunction [15]. In the following section, we briefly discuss the role of sleep, including NREM-sleep, REM-sleep and REM-dreaming in the modulation of emotion.

### 2.1. Sleep-deprivation affects emotional processing

Sleep loss and insomnia have been found to affect emotional reactivity and social function. Although the effects of sleep deprivation have been described at various levels of functioning, such as on the cognitive, psychomotoric or sensorimotoric level, the emotional effects might be most obvious in daily life functioning [16],[17]. Without enough healthy sleep, negative emotional reactivity seems to be significantly enhanced and positive reactions to positive events often subdued [17],[18]. A recent sleep deprivation study found that the response time for positive stimuli was faster than to negative and neutral stimuli, while accuracy in recognizing the valence of stimuli decreased after sleep deprivation [19].

In another recent sleep deprivation study, researchers analyzed changes in brain responses of 33 participants to a psychomotor vigilance task (PVT) during 13 fMRI sessions together with their subjective sleepiness report and positive and negative affect schedule (PANAS) report arranged in accordance with the circadian cycle, during 42 h of sleep-deprivation and after sleep recovery [20]. The results showed participants' negative affect remained relatively stable during the first day, then significantly worsened after the first and second melatonin onsets. These findings were also confirmed by Zohar and coworkers [18] who investigated the relationship between sleep loss and emotional reactivity in medical residents who were monitored for 5 to 7 d per 6 mo over a two-year period. The results showed that sleep loss not only intensified negative emotions, but even diminished positive emotions following a goal achieving event.

Deprivation or disruption of sleep is both a common symptom of and risk factor for a range of psychiatric disorders including anxiety and mood disorders. Also, in studies with children and adolescents it was found that sleep deprivation increased depression, confusion, and anger [21], as well as feelings of frustration and irritability/aggression [22]. Even after two nights of sleep deprivation, a significant increase in psychopathology scores have been found for bodily complaints, anxiety, depression and paranoia [23]. In another study from this research group [24], sleep deprivation was associated with an increase of extra-punitive responses, which reflects the tendency to show direct blame or hostility towards people or objects in the environment. Or in other words, it has been found that sleep deprivation leads to a decrease in accepting blame. These findings are in contrast with the effects of sleep deprivation on depression. Total or partial sleep deprivation, or selective restriction of REM-sleep in depression has showed that these acute sleep manipulations often lead to a temporary improvement in energy and mood, with a regression towards depression after any subsequent sleep [25]. The improvement of mood has been found even more strongly in ‘evening types’ assessed by the Hamilton depression rating (HDR) scale [26]. Without enough healthy sleep, including both NREM- and REM-sleep, negative emotional reactivity seems to be significantly enhanced, and positive reactions to positive events often decreased [26]. Sleep disturbances not only restrict our daily well-being and social functioning, but may even have a prognostic significance in the evolution of affective disorders like depression. Since NREM-sleep or REM-sleep serve to modulate emotional and motivational drives, these phases make the individual more emotionally flexible during wakefulness. Deprivation of especially REM-sleep for instance leads to more excitation in limbic brain structures resulting in enhanced emotional irritability and reactivity. After sleep deprivation, the normal regulation of the limbic system fails, resulting in an increased reactivity towards aversive emotional information. Along with the decrease in prefrontal activation, the regulation of emotions gets dysfunctional. Healthy sleep repairs adaptive processing and functional brain activity, integrity of the medial prefrontal cortex- amygdala connections important in emotion regulation processes [7],[10],[26].

Related to emotion regulation, relatively few studies to date, have directly addressed the impact of sleep on the spontaneous, habitual or dispositional tendency to use specific emotion regulation strategies or dispositional emotion regulation *via* the implementation of sleep deprivation [27]. In a study using event-related potential (ERP) to address the effects of sleep deprivation on the response to emotional pictures, they found that sleep-deprived individuals with lower reappraising capacity produced a larger late positive potential (LPP) response in response of negative pictures [28]. Apparently, after sleep deprivation, individuals with lower reappraisal ability show more difficulty in disengaging their attention away from negative stimuli. In future research, it should not only be important to take into account the individual's capacity of emotion regulation, but also to investigate how we can induce and train emotion regulation to influence sleep, as well as which sleep period should be most crucial for us to maintain a stable emotional state in the coming day. In the following section, we summarized NREM-sleep, REM-sleep and REM dreaming in relation to the modulation of emotion or emotion regulation.

### 2.2. REM-dreaming as emotion modulatory

REM-dreaming plays a crucial role in modulating people's emotions. In a review, Payne & Nadel [29] concluded that dream content varies as a function of sleep stage and time of night. Dreams seem to be more vivid and emotionally colorful during REM-sleep in comparison with dreams in other sleep stages where they have been found more of a thought like cognitive nature [30]–[32]. In REM-sleep, mentation has been found to be more expressive of motives and emotions [33],[34]. Moreover, the product of this culmination of emotions may result in nightmares during REM-sleep and NREM-sleep [35], in which negative emotional experiences are decreased, resulting in favorable “therapeutic” outcomes and compensating effects of emotion regulation failure in the daytime [36]–[38].

In another study, Cartwright et al. [39] did similar research on a clinically non-depressed group of community college students. Before and after each night of sleep, they filled out a mood scale. The second night they were woken up after several minutes of each REM-period and asked to report what they had been experiencing. The students who were more depressed showed better mood after dreaming. Cartwright concluded that depressive mood correlates with negative dream content, and that the modulatory function of REM-sleep dreaming appears when negative mood is moderate [26]. Similarly, in a study by Foulkes [30], when subjects reported their dreams spontaneously, they found a bias towards the negative or unpleasant content in their dreams. When the reporting was delayed, subjects especially recalled the more dramatic and emotionally anxiety charged contents. Conversely, in a study of Fosse et al. [40], emotion in sleep was assessed using instrumental awakenings. Subjects themselves had to score reported dream segments for the presence of negative and positive emotions. Combining this first-person rating with instrumental awakenings from REM-sleep, they found more positive emotions in comparison to negative emotions [41]. In contrast, other studies using this method of instrumental awakening reported more negative emotion such as anger and fear [42],[43]. Even when we take into account that dream reports are always given in waking state and therefore are constrained by the current state of the person, or group characteristics, we notice a clear tendency towards a predominance of negative emotion reported in most studies. This observation is further supported by the threat stimulation theory proposed by Revonsuo [44], which suggests that REM-dreaming repeatedly facilitates the rehearsal or the re-processing of the threatening events supporting more efficient threat perception and avoidance. Moreover, Levin and Nielsen [37] proposed in their review that the neurophysiological and neurobiological states characterizing REM dreams, in favor of essential cognitive processes, facilitates the expression of emotions and further allow a down-regulation of arousing emotions [45]. During REM-sleep, significantly increased activity in emotion-related areas, which includes subcortical areas (amygdala, striatum and hippocampus) and cortical areas (medial prefrontal cortex, mPFC) are paralleled by alterations in neurochemistry, with the most noticeable reduction in levels of noradrenaline, which is related to numerous arousal related emotion processing within the brain and the body [46],[47]. Specifically, the reactivation of the medial prefrontal cortex is probably functional in the regulatory and modulatory function of REM-sleep. In sum, the engagement of the amygdala and the cingulate cortex in the regulation of painful emotions during the day or during REM-sleep becomes especially modulated and regulated by the medial prefrontal cortex while it exerts a top-down regulatory function of limbic functioning [48]. REM-sleep may be adaptive to process aversive experiences such as traumatic experiences, by presenting them as strange images and fragmented episodes of related or similar stories [26]. In addition, strong evidence demonstrating the role of REM-sleep in regulating emotion comes from sleep modulation of conditional fear. A recent study found that REM-sleep during a daytime nap, as opposed to a nap without REM-sleep, reversed the progressive enhancement in experiences of fear by enhancing ratings of positive stimuli, suggesting an emotion regulatory function of REM-sleep [49]. In addition, research focusing on the impact of sleep on conditional fear response is based on the comparison between a night of sleep and the absence of intervening sleep, with the results suggesting that sleep, especially REM-sleep, consolidates conditional fear memories, allowing for an improved ability the next day to discriminate between threatening and non-threatening stimuli [50]. In addition to strengthening fear memories, sleep also adaptively facilitates the subsequent extinction of conditional fear, by way of top-town PFC inhibition of the limbic system. More interestingly, the extent of the fear extinction was found to be correlated with the amount of REM-sleep [51],[52]. Moreover, research evidence showed that sleep deprivation impairs the consolidation and extinction of fear memory due to a loss of top-down control from prefrontal cortex to subcortical limbic regions [48]. In summary, the above mentioned research indicates that the possible function of REM-dreaming in processing emotions is to re-process the emotional events experienced during the daytime, leading to the rehearsal of the possible emotion regulatory process of the brain which diminishes or adapts the impact of their emotional load on the activities of the following day in a way that is beneficial for the functioning during the day.

One of the most important theories for explaining the role of REM-sleep in modifying the emotional tone of previous experiences or memories is proposed by Walker and Van der Helm [38], which argued that REM-sleep acts as a state where the emotional tone is “depotentiated”, also known as the “sleep to remember, sleep to forget” theory. Humans sleep to forget the emotional tone, but still remember the tagged memory of the episode.

In this way, sleep (mainly REM sleep) functions as a way of decoupling the emotional tone from the emotional memories. However, several recent studies are challenging this “depotentiated” theory. For instance, Werner et al. [53] proposed that REM-sleep may not work as proposed by Walker, but instead maintain that the emotional tone of memories reinforces the emotional salience of events. Moreover, Hutchison et al. [54] and Genzel et al. [55] proposed that REM-sleep plays a role in the identification of the optimal outcomes for each specific memory (preservation or reduction of emotional tone), depending on the context as well as on the salience of the event. Whether REM-sleep plays a role in the maintenance of the emotional tone or the depotentiation of the emotional tones thus requires extensive further research.

### 2.3. NREM-sleep as emotion regulatory?

In the realm of emotion and sleep, research studies are mainly focused on the role of REM-sleep in processing emotions, leaving the role of NREM-sleep largely unknown. However, recent years have witnessed increasing research interest examining the importance of NREM-sleep in facilitating both fear extinction and emotion processing. For instance, by using olfactory contextual fear conditioning, it has been found that re-exposure to the odorant context in SWS promoted stimulus-specific fear extinction. This fear extinction process was supported by reductions of hippocampal activity and reorganization of amygdala ensemble patterns [56].

Another study using auditory contextual fear conditioning both during SWS and during wakefulness, however, suggested that the cued memory reactivation during SWS reinstated the fear response, whereas reactivation during wakefulness prevented the return of fear on the day of the study [57].

Furthermore, the fear extinction facilitation effect during SWS was further supported by He et al. [58], suggesting that NREM-sleep plays a key role in emotional memory processing, especially fear extinction [59],[60]. the above mentioned role of REM-sleep in consolidating emotional contents has recently been challenged. For instance, several recent papers have shown no effect of REM-sleep in selectively facilitating the consolidation of emotional memories.

In a recent study, examining the role of an afternoon nap in selectively consolidating negative aspects of scenes, it was found that NREM delta activity and the amount of SWS during the nap were robustly related to the selective consolidation of negative information [61]. Moreover, Cellini and their colleagues have found out that a daytime nap, regardless of whether it contains REM-sleep or not, facilitated the consolidation of declarative memories presented before and after sleep, irrespective of their valence [62].

## 3. Emotion, stress and sleep

As sleep is quintessential for our mood, mental health and daily well-being, the focus of the following section will be on the impairing effects of emotional stress on sleep.

### 3.1. Daily life and sleep

Daily life events influence both the general sleep physiology and affects dream patterns, as well as dream content and the emotion within a dream. In relation to how daily life events influence our sleep physiology, abundant research has focused mainly on the influence of pathological mood on sleep disturbance [7],[63],[64]. Understanding how normal variations in daily emotional experiences induce the changes in sleep can shed light on the vulnerabilities that facilitate the evolution of affect and sleep disorders. By conducting a two-week daily sampling approach to examine the impact of day-to-day variations in positive and negative emotion on nightly self-reported sleep-onset latency, sleep duration and sleep quality in young women, it has been confirmed that both positive and negative emotions experienced in daily life correlate with sleep [65]. A recent review [7] summarized that negative affective states, such as loneliness, grief or hostility, are found to be related with increased sleep impairments. Positive emotion, on the other hand, such as romantic love was found to be associated with decreased sleep duration and enhanced subjective sleep quality. It is worth mentioning that there is a lack of evidence about the effects of intense positive emotion such as feeling love, joy, and happiness on objective sleep quality, which also requires future research attention.

### 3.2. Emotional stress and sleep

Increased sleep fragmentation was found on the night before an exam [66],[67] and on the night before an operation [68]. Watching disturbing films before sleep has been reported to influence emotional experience in the first REM-periods of the night [69]. Furthermore, Åkerstedt and colleagues [70] found strong links between stress deriving from the social situation at work and impaired sleep. Another study also showed associations between interpersonal conflict and increased negative affect on the one hand, and increased sleep disturbance during that night, on the other hand [71]. Other social problems such as reduced social support and increased avoidance patterns usually result in psychological distress and sleep complaints [72],[73]. Moreover, women in the middle of a divorce proceeding displayed shorter REM-latency, higher percentage of total REM-sleep and decrease in SWS compared to a control group [74].

In another study on the effects of low, high and intermediate stress (over three nights within subjects) on sleep, participants have to rate their level of stress or worries at bedtime. Only those participants who differed in their level of stress or worries between nights were analyzed. Results showed a decrease in sleep efficiency, higher percentage of waking incidents and an increased latency to SWS [75]. Another study on pre-sleep negative affect resulted in reduced SWS, longer latency to SWS, more awakenings and less REM-sleep [76]. Also, Germain and associates [77] induced an acute stress exposure by telling subjects that they had to give a speech in the morning and that their performance would be evaluated. This resulted in an increase in REM-sleep density (REM-sleep density refers to the frequency of eye movements per unit of time during REM sleep) across REM-periods, a decrease in late-night average REM-count and a slower rate of increase across successive REM-periods immediately after the stress exposure. The increase in average REM-density is in line with previous studies on the effects of acute stress exposure [78]–[80]. Recently, Talamini and associates [81] found that emotional distress induced by a negative film before sleep, resulted in mild sleep deterioration, but also an increase in the proportion of SWS and increased number of REM-sleep-periods. In addition, emotional distress flattened this distribution and correlated with an increased number of REM-periods; the SWS-responses were positively correlated with emotional attenuation during sleep. However, still other studies have shown an inconsistent association between acute stress exposure and decreased ratio of REM-sleep and decreased REM-sleep duration [81],[82]. In a study of our sleep lab [83] the impact of an emotional failure experience on sleep physiology revealed a significant decreased sleep efficiency, increased sleep onset latency, wake after sleep onset, total time awake, number of awakenings during the night, number of awakenings from REM-sleep and a decreased % of REM-sleep, and SWS. In sum, research found that the effects of acute stress exposure on sleep in healthy persons resulted in more REM- but also NREM-sleep alterations. Pre-sleep stress can influence diverse aspects of sleep such as exaggerated startle response, decreased dream recall and elevated awakening thresholds from REM-sleep increased- or decreased-latency to REM-sleep, percentage of REM-density, REM-sleep duration and occurrence of arousals in sleep as a marker of sleep disruption, decrease in sleep efficiency, a high percentage of wake, increased latency to SWS and increased proportion of SWS [83]–[85].

In addition, the stressful events we experience in daily life appear to affect the content and emotions in our dreams. For example, research indicated that our daily variations in emotional pressure, distress and a disposition of experiencing highly reactive emotions resulted in greater nightmare prevalence, frequency, severity and in greater psychopathological comorbidity [75]. Also, the association between arousal and disrupted sleep in persons with insomnia suggests a strong relationship between daytime events and sleep disturbances [7]. In particular, REM-sleep alterations or abnormalities have been shown to be related to variables associated with the affective state of individuals during the day [86],[87]. Furthermore, research also indicates that children who have been exposed to stress during the early years of life develop adult insomnia more easily. Epigenetics in maladaptive stress responses in the new born ultimately predisposes the development of insomnia [78],[88].

For instance, during REM-sleep, there is a diminished functioning of the working memory and executive function circuit, combined with an enhanced adaptive functioning of brain networks subserving emotional processes and emotion regulation. REM-sleep after stress may thus function as a regulatory mechanism of waking emotional arousal [7]. Therefore, the adaptive influence of REM-sleep on the regulation of emotion appears to be reduced after REM-sleep deprivation in comparison with non-REM-deprived or undisturbed sleep [10],[26]. REM-sleep-dreaming functions as a central phase of the masked or unmasked reactivation and the reprocessing of emotions and emotional occurrences during the day. Dream production, especially in REM-sleep, which contains vivid simulations of painful and threatening events within real life, facilitates the processing of distressing emotions. Even more, it has been assumed to have a role in integrating traumatic and other distressing moments into our long-term memory. Changes in motivation and emotion are aspects in mood disorders, which are related with alterations in limbic processing not only during daytime, but also during REM-sleep. Every reprocessing of negative stressful events visually appearing in altered visualizations of what has happened in the day in dreaming may function to integrate negative experiences into long term memory in order to be prepared for future negative experiences.

Earlier REM-sleep onset and higher dream activity in the initial REM-period, have even been found to predict a greater reduction of the depressive symptoms after a distressing life event. It aids the processing of negative information making aversive events bearable. In particular, intensification of phasic REM-sleep appears to be a marker of dysfunctional or too little emotion regulation during the day indicating the need of further emotion processing and emotion regulation during sleep.

In summary, abundant evidence confirms a relationship between the emotional events we experience during the day and changed sleep physiology. Although consistent knowledge about stress can elicit profound and lasting effects on sleep, the pathways in stress affecting sleep are not well understood. Some research evidence has showed how the medial prefrontal cortex might play an important role in easing the detrimental effects of stress on sleep via the mediating activity of brain areas involved in the stress response such as the hypothalamus, the locus coeruleus and the limbic system [89]. Looking at the discussed findings, we notice an enhanced effective emotional adjustment and amelioration of mood and well being after intact sleep, especially REM-sleep. Good sleep may work as a bio-behavioral regulatory and restorative process that regulates daily emotional experiences and allostatic load of emotional stress [23]. Future research should pay attention to the pathways in which stress affects sleep. Of importance is how affected sleep physiology exerts further influence on following emotion processing.

## 4. Mediating role of trained emotion regulation

Importantly, recent research findings pointed out that emotion regulation, including an individual's - coping style and emotion regulation strategy may modulate the effects of emotional stress on sleep and especially REM-sleep [90],[91]. People may not always be able to control the stress factors they encounter in life, but they may be capable of adopting more efficient emotion regulation styles and thus control its detrimental influences on their sleep activity. In this way, people are able to avoid a “vicious cycle”, in which sleep deprivation compromises emotion regulation and results in increased negative emotion, which in turn disrupts sleep, leading to further impairments in emotional well-being. Sleep deprivation diminishes the capacity to regulate emotion. Racine and co-workers [92], for instance, found in a population of 523 women that those who slept less or more than population average 6-8 h sleep time were associated with decreased capacity of emotion regulation during sleep and subsequent day. Women, sleeping 6-8 h seemed to be more able to regulate their emotions in their waking lives. A longitudinal study of Tavernier and Willoughby [93] on 942 university students also showed that emotion regulation mediates the relationship between sleep problems and less positive social ties. In a study of Harvey [94], participants with insomnia used more suppression as emotion regulation as strategy and worried more than those with healthy sleepers who employed social control, replacement and reappraisal strategies. Also, the sleep of individuals prone to rumination may be significantly more negatively affected by stressful situations, resulting in a delayed sleep onset latency [94]–[96]. As discussed before, individuals with lower reappraisal capacity experiencing sleep deprivation elicited a larger late positive potential (LPP) in response to negative pictures [28]. In general, difficulties in emotion regulation correlate with rumination specific to insomnia, including unhelpful sleep-related thoughts and insomnia-related meta-cognition [97]. Prior research has mostly focused on the traditional cognitive strategies of controlling emotion regulation, such as reappraisal and suppression and less on more experiential emotion regulation strategies, such as focusing on the bodily affective feeling and becoming aware and eventual expression of it.

In order to make up for this lack, we will discuss the research of our lab, investigating the effectiveness of experiential emotion regulation.

### 4.1. Emotion approach as experiential emotion regulation strategy

Throughout the existing literature, there has often been a mistaken comparison or an interchangeable usage between rumination and an emotional coping approach to handling stressful situations, as if sensitivity to our emotional experience and reactions are maladaptive or equals a rigid and negative thinking state [11],[98]. Morin et al. [99], for instance, report that people with insomnia not only ruminate more, but also rely more on emotion-oriented coping strategies and experience more pre-sleep arousal than good sleepers. It has indeed been a broadly accepted view that emotional approach coping, defined as having an orientation to emotions, valuing and experiencing them intensely, might be a potential risk for temporarily enhancing psychological distress [100],[101]. This term appears, however, to be misleading, whereby emotion-focused strategies have also been operationalized as including a variation of strategies such as cognitively focusing on the understanding of one's own emotional avoidance, or focusing on one's own emotions or venting emotions. In the past, some operationalization of emotion-focused coping has thus often been confounded with an excessive focus on negative emotion, distress, facilitating ruminative thought.

As a consequence, it can be expected that the effectiveness of emotion-focused coping depends on the particular operationalization of an emotion-focused strategy employed. In addition, the correlational nature of much of the data cannot be used to maintain causation, as is often done in research on emotion regulation and coping. More experientially operationalized ‘emotion focused’ strategies, such as repeatedly processing negative emotions associated with a painful event in an affective manner [11], for instance, have been found to promote efficient recovery from and memory of emotionally negative situations [102]. Awareness of affective self-related material, when accompanied by experiential or bodily felt feeling is considered to be more effective in facilitating change than an overall cognitive focus [9]. As a consequence, evidence has shown that it is important in the processing of painful affect to move from the cognitive and analytical levels to the level of immediate experience, including emotional feelings. Depending thus upon the operationalization, some studies indicate that an emotion-approach coping style might indeed induce a reduction in total sleep time (as assessed using actigraphy) and a higher-level bedtime arousal [102]–[104]. On the other hand, it has been found that an emotion or experiential approach may in some case promote sleep [105]–[109]. In a study conducted by Vandekerckhove et al. [106] for instance, participants who were instructed to focus on their low level and concrete affective experiencing by affectively acknowledging, understanding their current affective feeling about a situation and to express it in an open non-evaluative mode, took longer time to fall asleep, but experienced significantly fewer awakenings, showed longer total sleep time, and higher sleep efficiency compared to the use of a cognitive analytical strategy where the participants were told to focus objectively and analyse their performance and its potential causes and consequences. In summary, the above studies suggested that although emotion focused coping or an experiential approach of an emotional stressor prior to bed time might make it more difficult to fall asleep, it might help regulating emotions to a greater extent, so that they are less likely to disrupt sleep during the night, leading to increased sleep duration, reduced sleep fragmentation and improved sleep quality.

Some research [107] highlighted the importance of properly adjusting one's coping strategy based on the perceived controllability of stressors. Specifically, in order to have a good sleep quality, it has already been shown that emotion approach appears to be more beneficial in situations when the stressor is uncontrollable (*e.g.*, the stress when waiting for the grade of the exam), whereas problem-focused coping appears to be more beneficial than emotion-approach coping in dealing with controlled stressors (*e.g.*, the stress from an upcoming exam). This point of view was further supported and highlighted in reviews addressing how emotion, ER and sleep interact with each other [26],[66],[108],[109].

## 5. Conclusion

In this review, we illustrate the intimate relationship between sleep and emotion in various ways, especially emphasizing the modulating function of sleep on daily emotion and the modulating role of emotion regulation on the interplay between emotional stress and sleep. As a matter of daily experience and empirical evidence, emotion and emotion regulation play an important role in the interplay between stress and sleep disturbance. Intact sleep is crucial for our general well-being, considering that NREM-sleep, REM-sleep and REM-dreaming play a modulating function on our emotions experienced during the day. During REM-sleep, the diminished functioning of the executive function circuit combined with an enhanced adaptive activity of limbic networks which support emotional processes helps us to regulate the emotional events that we encounter during daily life. For instance, morning mood improves when REM-sleep is intact, but worsens after a night of sleep deprivation. In line with these findings, dreaming may be functional to process negative emotional experiences. Even more, it has been assumed to have a role in integrating traumatic and other distressing memories into memory. Sleep disturbances not only restrict our daily well-being and social functioning, but even have a prognostic meaning in the evolution of affective disorders like depression. Sleep deprivation is characterized by an increased reactivity towards aversive emotional information. Together with the decrease in medial prefrontal activation, the capacity for emotion regulation becomes dysfunctional. Healthy sleep repairs adaptive processing, functional brain activity, integrity of the medial prefrontal cortex-amygdala connections, and thus improves the capacity to regulate emotions as well as an individuals' well-being.

Research findings confirm the common sense view that stressful events and acute stress as well as our way of coping with it have an impact on sleep. Despite a long history of interest in the mechanisms that regulate sleep such as emotion regulation as well as the role of sleep in impacting emotions that we experience during the day, the relationship between our emotional brain and sleep is still a neglected area of research. Therefore, research and reviews on emotion-sleep interaction and emotion regulation is necessary to help us further understand specific dispositional and situational functional characteristics of the brain before and during sleep, as well as its proposed role in the regulation of emotions in daily life. Psychophysiological factors such as stress, anxiety, and hyperarousal, play an important role in causing sleep disturbances. Also, sleep disturbance predicts the subsequent development of mental health, while the development of insomnia predicts psychopathology such as depression or post-traumatic stress disorder after an acute stressful event. In the meanwhile, insomnia sufferers report more difficulty coping with daily stressors than good sleepers. This finding has led to the suggestion that it is especially the specific individual's response and way of coping with it that determines the level of emotional stress an individual experience after a painful event. Although the mechanisms that regulate sleep have recently been subjected to detailed investigation, the role of emotion regulation in emotion processing, subsequent sleep within the sensitivity for positive and negative affect -especially the sustainment of negative affect- is still an important domain to further investigate. Until now, it has been found that the effectiveness of emotion-focused coping strategies depends on the particular operationalization of the emotion-focused strategy employed.

In the past, emotion-focused coping has often been confounded with an excessive focus on the understanding of the negative emotion itself, as well as with emotional avoidance, distress and venting emotions, which might enhance arousal and facilitate ruminative thought increasing sleep problems. On the other hand, experiential ‘affective or ‘emotion focused’ strategies, defined by coping through affectively acknowledging, understanding, and expressing actual emotional experience and affective feeling about a situation has been proven to facilitate efficient recovery from as well as memory of emotionally negative situations with positive effects on sleep. Focusing on the awareness of affective self-related material, when accompanied by experiential or bodily awareness of a feeling is considered to be more effective in facilitating change than an overall cognitive focus.

In summary, increasing evidence confirms that it is crucial to the processing of painful affect to move from the cognitive and analytical level to the level of immediate experience, including emotional feelings. Also, some studies have demonstrated that a tendency to emotionally approach the stressor may disrupt sleep, whereas an experiential operationalized emotion regulation may promote sleep. Considering the influential role of emotion regulation strategies in easing the detrimental effects of stressful events on subsequent sleep, further research has to clarify which emotion regulation strategy in which situation can modulate sleep in a most beneficial way. In this context, considering the influential role of emotion regulation strategies in easing the detrimental effects of stressful events on subsequent sleep, we call for further research to clarify the possible pathways through which emotion regulation can modulate sleep and to explore which strategy is the most effective in the regulation of emotions before sleep.
